# Fabrication of Piezo-Resistance Composites Containing Thermoplastic Polyurethane/Hybrid Filler Using 3D Printing

**DOI:** 10.3390/s21206813

**Published:** 2021-10-13

**Authors:** Kyoungho Song, Hansol Son, Suwon Park, Jonghan Lee, Jungsik Jang, Mijung Lee, Hyun-joo Choi

**Affiliations:** Department of Materials Science and Engineering, Kookmin University, Seoul 02707, Korea; 4byeol@kookmin.ac.kr (K.S.); tjseodkr@kookmin.ac.kr (H.S.); tndnjs211@kookmin.ac.kr (S.P.); ljh06_19@kookmin.ac.kr (J.L.); kmjanggo@kookmin.ac.kr (J.J.); mijung@kookmin.ac.kr (M.L.)

**Keywords:** conductive composites, 3D printing, hybrid filler, piezoresistive sensitivity, health monitoring, touch/flexible sensors

## Abstract

In this study, 3D-printable flexible piezoresistive composites containing various amounts of cilia-like hybrid fillers were developed. In the hybrid fillers, micro-scale Cu particles with a 0D structure may allow them to easily disperse into the flexible TPU matrix. Furthermore, nanoscale multi-walled carbon nanotubes (MWCNTs) with a high aspect ratio, present on the surface of the Cu particles, form an electrical network when the polymer matrix is strained, thus providing good piezoresistive performance as well as good flowability of the composite materials. With an optimal hybrid filler content (17.5 vol.%), the 3D-printed piezoresistive composite exhibits a gauge factor of 6.04, strain range of over 20%, and durability of over 100 cycles. These results highlight the potential applications of piezoresistive pressure sensors for health monitoring, touch sensors, and electronic skin.

## 1. Introduction

Smart fashion has generated considerable interest in various fields such as commerce, medicine, military, and aerospace. Among wearable technologies, flexible piezoresistive materials have potential applications in strain/pressure sensors, touch sensors, electronic skin, and biomedical robotics [1].

One way to produce flexible piezoresistive materials is to utilize conductive polymer composites (CPCs), wherein a conductive network is formed inside the material during its deformation and, when a larger external force is applied, a change in the electrical response occurs. In the past few years, flexible CPCs with various fillers (i.e., metallic particles, conductive polymers, and carbon materials) have been reported. For example, several studies have reported an improvement in electrical conductivity by dispersing metallic pillars such as copper, gold, and silver in the form of nanoparticles, nanowires, and nanosheets [2]. However, the poor compatibility of the interface between the metallic fillers and polymer matrix can adversely affect the piezoresistive properties. Thus, owing to their compatibility with the matrix, the use of conductive polymers as fillers has been proposed by few researchers. Conductive polymer fillers such as poly(3,4-ethylenedioxythiophene): poly (styrene-sulfonate) (PEDOT: PSS), poly(styrene-β-isobutylene-β-styrene), and poly (3-hexylthiophene) are widely used to obtain stretchable and cost-efficient composites using various methods, such as chemical doping and dip coating [3,4,5]. Although PEDOT: PSS is well known as a suitable conductive polymer for a filler, its electrical conductivity (4380 S cm^−1^) is far lower than that of metallic or carbon-containing filler composites (5.96 × 10^7^ S cm^−1^ for copper and 10^6^ to 10^7^ S cm^−1^ for CNTs) [6]. Another candidate for CPCs is carbon nanofiller, which shows no delamination owing to its better compatibility compared to that of metal fillers and has a high electronic conductivity. Several studies have reported the use of carbon nanofillers with high aspect ratios (i.e., width-to-thickness ratios) to form conductive pathways in the insulating polymer matrix. These studies have demonstrated that polymer/CNTs or polymer/graphene composites have good piezoresistive performances [6,7,8,9,10,11,12]. However, carbon fillers generally tend to agglomerate owing to their affinity resulting from strong Van der Waals forces; this issue adversely affects the uniform dispersion of the carbon filler in the flexible matrix. Recently, many attempts have been made to fabricate polymer composites using a variety of processes, especially in the field of functional composite materials. For instance, using the chemical reaction of polyhedral oligomeric silsesquioxane (POSS) and halloysite nanotubes (HNTs), Wu et al. suggested that the inclusion of HNTs-POSS enhances the fire safety of TPU nanocomposites [10]. In addition, one recent study shows that polyvinylidene fluoride (PVDF) films containing graphene nanoplatelets (GNPs) are suitable for flexible strain sensor fabrication using the solution mixing method [11]. Recently, graphene-based piezoresistive materials with a 3D structure have been synthesized by various methods such as 3D printing, self-assembly, and electrospinning [12]. Although 3D structure has been known to greatly enhance sensor performance, the low-cost, large-scale synthesis of 3D-structured piezoresistive materials is still challenging.

Herein, to address the agglomeration problem of carbon nanomaterials in polymer matrix, we propose a new class of flexible piezoresistive materials containing MWCNTs/metal hybrid fillers. In our previous study, a cilia-like metal/CNTs hybrid powder was proposed that formed a 3D structure and exhibited good electrical conductivity in the polymer matrix [13]. In piezoresistive materials, hybrid fillers may form geometrically conductive networks when the polymer matrix is strained. This study describes the use of an additive manufacturing technique to enhance the design flexibility of the newly developed piezoresistive materials. Subsequently, the piezoresistive performance of the 3D-printed flexible composites containing monolithic and hybrid fillers is discussed with respect to the microstructures and electrical conductivities.

## 2. Experimental

### 2.1. Materials and Methods

**Figure 1:**
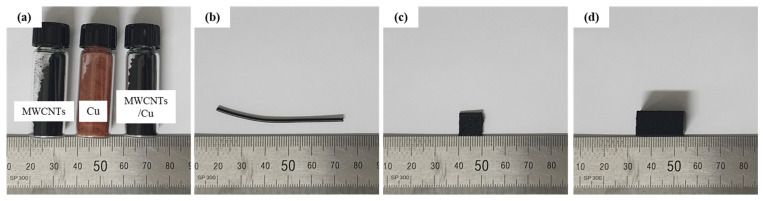
(**a**) Raw material images and the hybrid powder after mechanical milling; (**b**) extruded filaments of TPU/Cu/MWCNT composites before 3D printing; and the 3D-printed test specimen for (**c**) interrogation of the electrical conductivity and (**d**) compression test.

Cu/MWCNT hybrid powder was fabricated by mechanical milling of a mixture of Cu powder (Cu, mean size 1 μm (±1.25), spherical shape; Kojundo Co. Ltd., Japan) and MWCNTs (diameter ~20 nm, length ~10 μm, purity > 90%; Applied Carbon Nano Technology Co. Ltd., Korea). The Cu and MWCNTs powders, as well as the ball-milled composite powder, are shown in Figure 1a. A planetary mill (Pulverisette 5; FRITSCH Inc., Germany) was used to uniformly disperse and partially insert MWCNTs on the surface of the micro-sized Cu particles. The weight ratio of the MWCNTs to Cu powder was 1:2. The milling process was conducted for 2 h at a milling speed of 200 rpm and a powder-to-ball weight ratio of 1:15. The composite powder was then mixed with thermoplastic polyurethane (TPU, round shape pellet; Shandong Huada Chemical New Material Co., Ltd., China) to produce pellets for 3D printing. TPU was dissolved in N,N-dimethylformamide (DMF) solution (Daejung Chem. Co. Ltd., Korea), and the Cu/MWCNT hybrid powder was added to the TPU polymer solution and mixed using a magnetic stirrer for 4 h at room temperature to obtain a well-dispersed TPU/Cu/MWCNT solution. The solution was dried and then degassed in a solvent hood for 24 h to remove DMF. The volume fractions of the filler (i.e., Cu/MWCNTs) were 5, 15, 17.5, and 20%. The TPU/Cu/MWCNT composite was cut to fabricate a filament for the 3D printer, as shown in Figure 1b. The chopped TPU/Cu/MWCNTs were then extruded using a mini-extruder (Filabot EX2, Filabot Co., USA) with a diameter of 1.75 mm where the melting temperature was in the range of 180–210 °C. We prepared two types of 3D-printed composites to measure the electrical conductivity (Figure 1c, 10×10×1.1 mm) and conducted an experiment for investigating the piezoresistive behavior (Figure 1d, 9.5×9.5×19 mm). To prepare the 3D-printed TPU/Cu/MWCNT composites, the filaments were 3D printed (fused deposition modeling) using a commercial 3D printer (3DP; 3DP-310FB, HyVISION SYSTEM Inc., Seongnam, Korea). The processing parameters of the printed TPU/Cu/MWCNTs are listed in Table 1.

### 2.2. Morphological, Electrical and Piezoresistive Characterization

For microstructural morphology evaluations, a field emission scanning electron microscope (FE-SEM; JEM-7410F and JEM-7610f, JEOL Ltd., Japan) was used to observe the morphology of the Cu/MWCNT hybrid particle. An optical microscope (OM, Daemyung Co. Ltd., Korea) was used to observe the cross-sectional microstructure of the TPU/Cu/MWCNT hybrid filaments. Raman spectroscopy (Raman Spectrometer, LabRam ARAMIS, Horiba, Japan) was performed in the range of 1000–3000 cm^−1^. The 4-point-probe method was used to measure the sheet resistance of the 3D-printed TPU/Cu/MWCNT composites. The piezoresistive performance was tested using a universal tensile machine (UTM, RB 301 UNITECH-M, R&B Inc., Daejeon, Korea) and a multimeter (DM3058E, RIGOL Technologies, Japan) to measure the change in the electrical resistance with respect to the applied tension. For this test, the 3D-printed compression specimen was loaded with a crosshead speed of 1 mm/min using a 1-ton load cell. Copper tape (10 cm) was attached to the top and bottom sides of the 3D-printed compression specimen, which acted as electrodes. A 100× cyclic compress test was conducted under the same conditions as the compression test at displacements of 1 and 1.5 mm to illustrate the persistence of the piezoresistive performance.

## 3. Results and Discussion

### 3.1. Surface and Cross-Sectional Morphology

**Figure 2:**
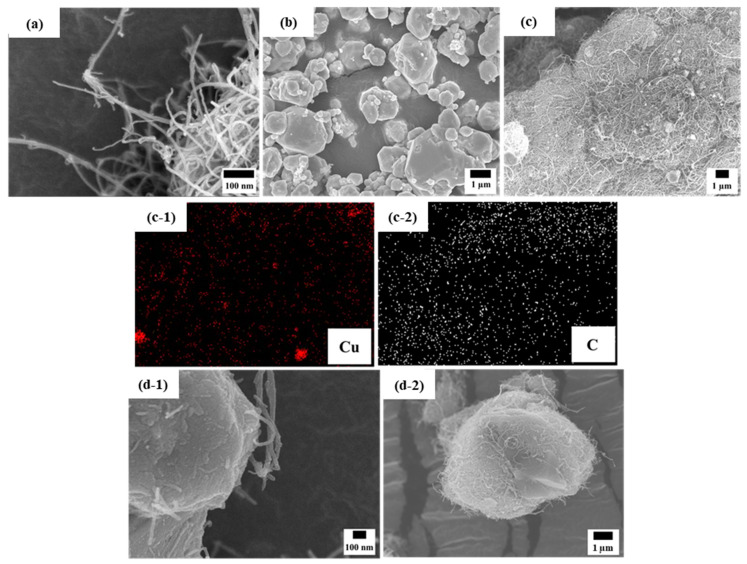
FE-SEM images of (**a**) initial MWCNTs, (**b**) initial Cu powder, (**c**) ball-milled hybrid powder and its corresponding EDS map (**c-1**,**c-2**), and (**d-1**,**d-2**) magnified images of the ball-milled hybrid powder wherein MWCNTs are partially embedded into Cu particles.

Figure 2 shows FE-SEM images of the MWCNTs, Cu powder, and ball-milled hybrid powder and its corresponding energy-dispersive spectrometer (EDS) map. The MWCNTs (Figure 2a) had an average diameter of 20 nm and were found to be curved and entangled. In contrast, the initial Cu powder was observed to be mostly spherical, but the sizes and shapes of the particles was irregular. The micro-sized Cu powder is difficult to plastically deform when mixed with entangled bundles of MWCNTs; therefore, the Cu powder was covered with MWCNTs, as shown in Figure 1c and its corresponding EDS map. As shown in Figure 2d-1,d-2, the hard and stiff MWCNTs partially embedded in the Cu powder and dispersed to some extent along with the plastic deformation of the Cu powder.

**Figure 3:**
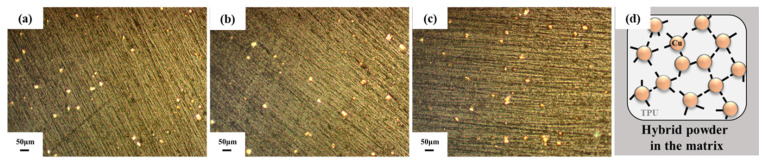
OM images of the TPU/Cu/CNTs filament cross section after extruding (**a**) 15, (**b**) 17.5, and (**c**) 20 vol.%; (**d**) schematic of the hybrid particles in the matrix.

The ball-milled Cu/MWCNT hybrid powder was then mixed with a flexible TPU; Figure 3a–c show optical images of the cross section of the hybrid filament. As shown, the bright particles (presumably the Cu/MWCNT hybrid powder) were well dispersed in the flexible TPU matrix. Because the bright Cu particles in the OM images seem to be uniformly dispersed in the TPU matrix, it is assumed that the nano-sized MWCNTs, attached to the surface of the Cu particle (as shown in Figure 2), were also uniformly dispersed in this filament. The nano-sized MWCNTs are not seen in these OM images because of the low magnification. As the Cu/MWCNT hybrid powder content increased, the number of bright particles was also observed to increase, and the distance between each particle gradually decreased. The micro-scale Cu particles with a 0D structure may allow them to easily disperse into the flexible TPU matrix relative to the dispersion of nanoscale MWCNTs with a high aspect ratio. However, the MWCNTs, which cannot be easily dispersed solely in TPU, can be readily dispersed with the help of Cu powder because they cover or are embedded in the Cu powder, as shown in the schematic depiction of the microstructure of Cu/MWNCT/TPU pallets in Figure 3d. Thus, it can be expected that the 1D structure (i.e., a high aspect ratio) of the MWCNTs plays a pivotal role in the formation of conductive pathways surmounting the difficulty of dispersion of the nano-sized and entangled MWCNTs.

**Figure 4:**
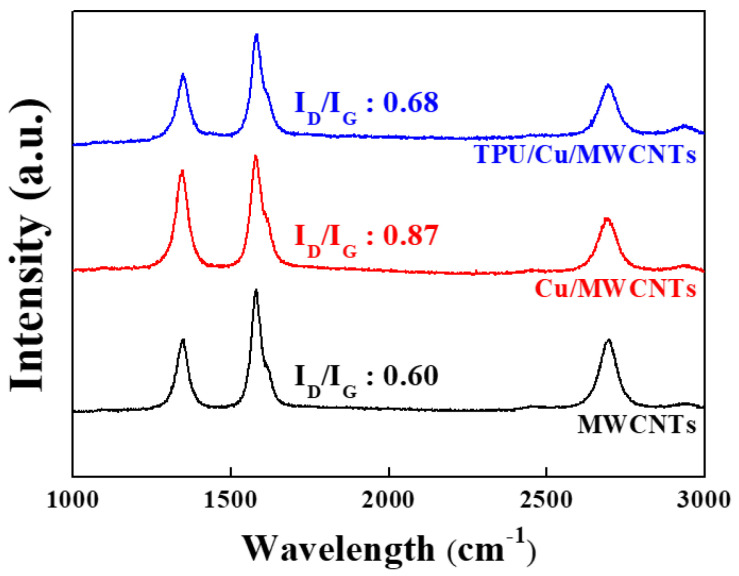
Polarized Raman spectra of TPU/Cu/MWCNTs, Cu/MWCNTs, and MWCNTs.

Raman spectra analysis is a useful method to examine the changes in the molecular structure of MWCNTs in the TPU matrix during mechanical milling and 3D printing processes. As shown in Figure 4, sharp and strong peaks appeared at ~1580 cm^−1^, which is in the G band, for all the samples, and its intensity was verified by the in-plane microstructure of the graphitic structure [14]. Other peaks at ~1350 cm^−1^ and ~1615 cm^−1^, which are in the D and D′ bands, respectively, were also observed for all the samples. The ratio of the intensity of the D peak to that of the G peak, which is used to infer the amount of structural defects in graphitic materials, increased from 0.60 to 0.87 after mechanical milling. The increase in the ratio of I_D_/I_G_ indicates that additional defects might have been created owing to mechanical milling. However, the I_D_/I_G_ ratio of 3D-printed TPU/Cu/MWCNT composites decreased to 0.68, which is comparable to that of the raw MWCNTs. During the extrusion and 3D printing, the specimens were exposed to high temperatures, which might have led to a recombination of the internal defects in the MWCNTs. Gong et al. have reported that the density of defects reduces during heat treatment [15]. Therefore, the molecular structure of MWCNTs is not significantly destroyed throughout the entire process.

### 3.2. Electrical and Piezoresistive Properties

**Figure 5:**
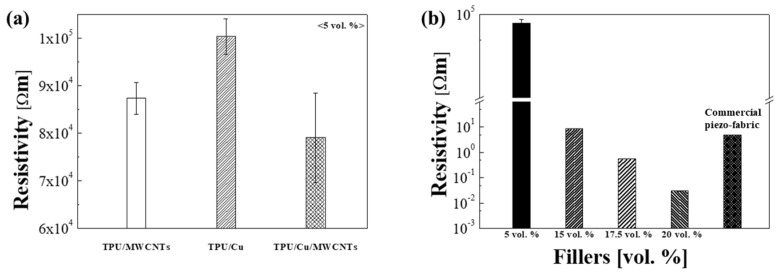
(**a**) Resistivity of the different fillers containing TPU at the same volume fraction; (**b**) resistivity of the 3D-printed TPU/Cu/MWCNTs compared with commercial piezoresistive fabric at different volume fractions.

As shown in Figure 5a, the electrical properties of the 3D-printed TPU/Cu/MWCNT hybrid composites were compared with those of TPU composites dispersed with either Cu or MWCNTs, wherein, in all the cases, the amount of the filler was fixed at 5 vol.%. The sheet resistivity of the TPU/Cu, TPU/MWCNTs, and TPU/Cu/MWCNT hybrid composites was $1.0\times 10^{5}\;Ωm$, $8.7\times 10^{4}\;Ωm$, and $7.9\times 10^{4}\;Ωm$, respectively. MWCNTs were found to be more effective than micro-sized Cu particles as conducting agents because the presence of nano-sized 1D MWCNTs with a high aspect ratio enables the formation of electron pathways, compared to micro-sized and spherical Cu. However, owing to the presence of Van der Waals forces, entangled MWCNTs cannot be easily dispersed in the TPU matrix. Because MWCNTs can be uniformly dispersed with the help of micro-sized Cu particles, the composites containing Cu/MWCNT hybrid fillers exhibited better electrical conductivity (lower sheet resistivity) than those containing solely dispersed MWCNTs. These Cu/MWCNT hybrid fillers may effectively form a conductive network. When the MWCNTs are uniformly dispersed, electrons may readily move from one MWCNT tip to another because of the tunneling effect. Figure 5b shows the resistivity of the 3D-printed TPU/Cu/MWCNTs with different volume fractions of Cu/MWCNT fillers, which was then used to determine the optimized volume fraction of the fillers required for 3D printing. The electrical resistivity of the specimens exponentially decreased with an increase in the volume fraction of the fillers, exhibiting a typical percolation threshold [16]. Referring to the OM image in Figure 3, it was deduced that when the distance between the hybrid particles in the TPU matrix decreased, the electrical conductivity was enhanced, owing to an increase in the conductive pathways such as electron tunneling. The electrical resistivity of the composites developed in the present study was also compared with that of a commercial piezoresistive pressure sensing fabric (EeonTex™ NW170-SLPA-2k, SparkFun Electronics, Niwot, CO, USA). When the filler content was greater than 15 vol.%, the resistivity was close to, or lower than, that of the commercial piezoresistive fabric. It is worth noticing that, along with the advantage of it being suitable for 3D printing, the new material developed in the present study shows superior electrical conductivity compared to that of commercial piezoresistive materials. This is important because the rheological properties of nano-composite materials are known to deteriorate during fused deposition modeling (FDM) type 3D printing.

**Figure 6:**
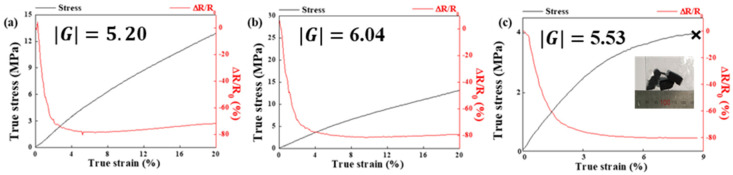
Normalized change of piezoresistive sensitivity and compressive stress versus strain for TPU/Cu/MWCNT composites at (**a**) 15 vol.%, (**b**) 17.5 vol.%, and (**c**) 20 vol.%.

The piezoresistive behavior of the 3D-printed hybrid composites was investigated via in situ compression tests, wherein the ratio of the initial resistivity (R_0_) and resistivity change (ΔR; ΔR = R−R_0_, where R is the resistivity at a given strain) was monitored in real time till the composites were strained up to 20% (ε≤20%, where ε is the true strain during compression) or fractured (fracture strain was 8.7%), and the results are shown in Figure 6a–c, respectively. The sensitivity of a piezoresistive material can be quantified through the gauge factor, G, using the following formula:

(1) $$G=\left(\Delta R/R_{0}\right)/\varepsilon \;\ldots$$

G can be defined as the slope of the ΔR/R_0_ curve during compression. For convenience of comparison, the absolute values of G were used in the present study. The applied compressive true stress and the ΔR/R_0_ values were plotted against the compressive true strain. The gauge factor in the strain range of 0–8.7% was calculated. The gauge factors of the TPU/Cu/MWCNT hybrid composites with filler contents of 15, 17.5, and 20 vol.% were measured as 5.20, 6.04, and 5.53, respectively. The ΔR/R_0_ curves show a similar tendency of exponentially decreasing piezoresistive sensitivity for all the specimens in the strain range up to 5%. During the initial stage of compression (strain < 5%), conducting pathways can be easily formed with deformation as conducting fillers get closer and assemble. Once the percolation threshold was reached, the piezoresistive sensitivity value saturated with further straining. In the present study, MWCNTs may help the Cu hybrid particles form continuous conductive networks under compressive strain. In terms of the value of the gauge factor, an applied deformation to the 17.5 vol.% sample was observed to induce strong and reversible variations in the conductive network configuration, which lead to a larger variation in the electrical conductivity. However, for composites with higher filler content (>20 vol.%), the piezoresistive sensitivity was observed to deteriorate, possibly because of the low sample quality (e.g., internal pores or defects) stemming from the low fluidity of the samples during printing. Furthermore, the disruption or modification of even a single conductive path may also deteriorate the piezoresistive sensitivity.

**Figure 7:**
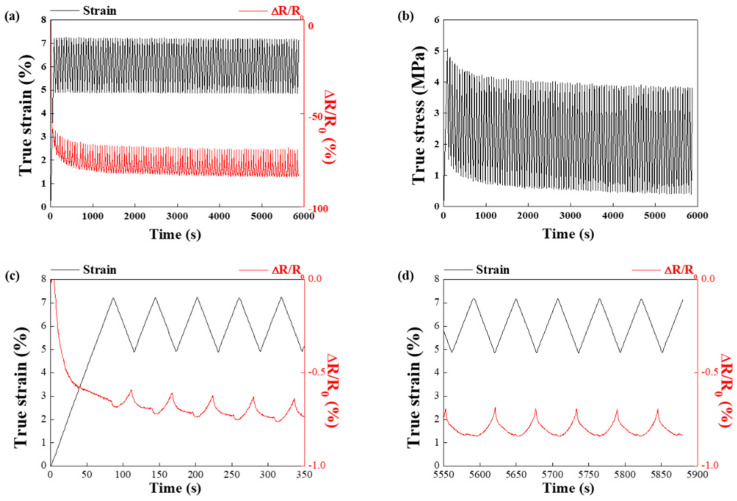
(**a**,**b**) Compression testing of 3D-printed TPU/Cu/MWCNT composites up to 100 cycles (**c**) first and (**d**) last 5 cycles.

The reproducibility of piezoresistive sensitivity to strain during 100 cycles was also tested for the 17.5 vol.% hybrid filler (Figure 7a,b). This cycling test was conducted in the compression strain range of 5–7% (1–1.5 mm displacement). For the first 10 cycles, the ∆R/R_0_ value reached approximately −60 to −80% in a linear fashion and did not recover to its initial value (Figure 7a); this may have occurred because of the rearrangement of the hybrid fillers in the flexible TPU matrix. When the ΔR/R_0_ value reached −60 to −80%, the change in amplitude of ΔR/R_0_ was insignificant, and the hybrid fillers formed a conductive network after rearrangement. The true stress decreased significantly for the first 10 cycles and then stabilized (Figure 7b), possibly because of the viscoelastic properties of the TPU matrix [17,18]. In the first 5 loading cycles of the composites containing hybrid fillers, ∆R/R0 initially decreased and subsequently increased (Figure 7c). In contrast, ∆R/R0 was observed to stabilize in the last 5 cycles (Figure 7d). During the initial loading stage, the reinforcement may rearrange to form a more effective electrical network; hence, the ∆R/R0 values may fluctuate. As reported elsewhere, the ∆R/R0 values can be stabilized once the filler network is stable [19].

## 4. Conclusions

When the filler content was greater than 15 vol.%, the resistivity was close to that of a commercial piezoresistive fabric. It is worth noticing that the new material developed in the present study shows superior electrical conductivity compared to that of commercial piezoresistive materials, along with it being suitable for 3D printing. This is important because the rheological properties of nano-composite materials are known to deteriorate during fused deposition modeling (FDM) type 3D printing.

In this study, flexible TPU-based 3D printable composites containing hybrid fillers, which consist of Cu covered or partially embedded with MWCNTs, were prepared using the solution mixing method. Composites containing 5 wt.% hybrid fillers exhibited higher electrical conductivity than those containing the same content of monolithic fillers, which demonstrates that hybrid fillers are beneficial for forming electrical networks. Furthermore, the hybrid fillers allow them to easily disperse into the flexible polymer due to its structural feature. Thus, owing to their microstructural characteristics, the 3D printable TPU/Cu/MWCNT composites showed better electrical conductivity than TPU composites dispersed with only Cu or MWCNTs. As the hybrid filler content increased, a higher gauge factor was obtained. However, the composites with the highest filler content (20 vol.%) showed rather deteriorated and lower gauge factors. In the case of the optimized composites (17.5 vol.%), the 3D-printed TPU/Cu/MWCNT composites showed a gauge factor of 6.04 and a strain range of over 20%. Furthermore, it is worth noticing that 3D printable composites containing the hybrid filler showed superior electrical conductivity than a commercial piezoresistive fabric. Finally, when using a proper amount of filler in the matrix during the 3D printing process, the TPU/Cu/MWCNT composites showed excellent electrical conductivity while maintaining flexibility and are thus a good candidate for electrical resistive materials in smart fashion.

## Figures and Tables

**Table 1:** 3D printing parameters to fabricate 3D-printed TPU/Cu/MWCNT composites.

Processing Parameter	Value
Print nozzle diameter	0.4 mm
Extruder temperature	210 °C
Bed temperature	80 °C
Chamber temperature	35 °C
Layer height	0.2 mm
